# Toll like receptor 4 contributes to blood pressure regulation and vascular contraction in spontaneously hypertensive rat

**DOI:** 10.1042/CS20110523

**Published:** 2012-06

**Authors:** Gisele F. Bomfim, Rosangela A. Dos Santos, Maria Aparecida Oliveira, Fernanda R. Giachini, Eliana H. Akamine, Rita C. Tostes, Zuleica B. Fortes, R. Clinton Webb, Maria Helena C. Carvalho

**Affiliations:** 1 Department of Pharmacology, University of Sao Paulo – Sao Paulo – SP – Brazil; 2 Department of Physiology, Georgia Health Sciences University – Augusta – GA – United States; 3 Department of Pharmacology, School of Medicine of Ribeirao Preto, University of Sao Paulo – Ribeirao Preto – SP – Brazil

**Keywords:** toll like receptor 4, hypertension, mesenteric resistance arteries, cyclooxygenase, inflammation

## Abstract

Activation of Toll-like receptors (TLR) induces gene expression of proteins involved in the immune system response. TLR4 has been implicated in the development and progression of cardiovascular diseases. Innate and adaptive immunity contribute to hypertension-associated end-organ damage, although the mechanism by which this occurs remains unclear. In the present study we hypothesize that inhibition of TLR4 decreases blood pressure and improves vascular contractility in resistance arteries from spontaneously hypertensive rats (SHR). TLR4 protein expression in mesenteric resistance arteries was higher in 15 weeks-old SHR than in same age Wistar controls or in 5 weeks-old SHR. In order to decrease activation of TLR4, 15 weeks-old SHR and Wistar rats were treated with anti-TLR4 antibody or non-specific IgG control antibody for 15 days (1µg per day, i.p.). Treatment with anti-TLR4 decreased mean arterial pressure as well as TLR4 protein expression in mesenteric resistance arteries and interleukin-6 (IL-6) serum levels from SHR when compared to SHR treated with IgG. No changes in these parameters were found in Wistar treated rats. Mesenteric resistance arteries from anti-TLR4-treated SHR exhibited decreased maximal contractile response to noradrenaline compared to IgG-treated-SHR. Inhibition of cyclooxygenase-1 (Cox) and Cox-2, enzymes related to inflammatory pathways, decreased noradrenaline responses only in mesenteric resistance arteries of SHR treated with IgG. Cox-2 expression and thromboxane A_2_ release were decreased in SHR treated with anti-TLR4 compared with IgG-treated-SHR. Our results suggest that TLR4 activation contributes to increased blood pressure, low grade inflammation and plays a role in the augmented vascular contractility displayed by SHR.

## Introduction

Toll like receptor 4 (TLR4) is expressed on virtually all human cells and binds a wide spectrum of exogenous (including bacterial lipopolysaccharide - LPS) and endogenous (heat shock protein, angiotensin II) ligands. TLR4 is involved in innate immune responses to various infectious agents and stressors.[1] In the presence of a ligand, the TLR4 receptor activates intracellular pathways that culminate in nuclear factor κ B (NFκB) phosphorylation resulting in an inflammatory process characterized mainly by the production of pro-inflammatory cytokines.[2]

TLR4 has been implicated in mediating chronic inflammatory diseases, including cardiovascular diseases.[3] TLR4 upregulation has been observed after myocardial infarction in the heart of mice[4] and it is also related to the initiation and progression of atherosclerosis.[5] TLR4 expression is augmented in cardiomyocytes from spontaneously hypertensive rats (SHR), when compared with Wistar Kyoto rats, suggesting that this receptor may be implicated in the hypertension-associated end-organ damage.[6]

One of the downstream products of TLR signaling is cyclooxygenase-2 (Cox-2) and pro-inflammatory cytokines.[7] Cox-2 is an inducible isoform of the cyclooxygenase enzyme that catalyzes the formation of prostaglandins, which may mediate inflammatory responses. One important prostaglandin produced by Cox-1 and Cox-2 is thromboxane A_2_ which is a potent vasoconstrictor that contributes to increased vascular contraction in arterial hypertension.[8]

Arterial hypertension is a well-known risk factor for various cardiovascular diseases and currently, it is estimated that 25% of the adult population is affected by this disease.[9] It has been suggested that both innate and adaptive immunity contribute to the pathophysiology of hypertension.[10] In this regard, Guzik and colleagues observed that mice lacking lymphocytes are resistant to the development of hypertension. Adoptive transfer of T cells restored hypertensive responses to angiotensin II and deoxycorticosterone (DOCA)-salt challenge in these mice [11], showing the importance of adaptive immune response in hypertension.

Considering the concept that inflammation is closely linked to hypertension and that the role of innate immune system, represented by TLR4 activation, in hypertension is still unclear, we hypothesized that TLR4 is up-regulated in SHR resistance arteries and its increased activation contributes to both increased vascular response to contractile stimuli and augmented blood pressure levels in SHR.

## Materials and Methods

### Animals

Male Wistar rats (15 weeks-old) and SHR (5 and 15 weeks-old; Harlan Laboratories, Indianapolis, IN, USA) were used in this study. All procedures were performed in accordance with the Guiding Principles in the Care and Use of Animals, approved by the Medical College of Georgia Committee on the Use of Animals in Research and Education and in accordance with the Guide for the Care and Use of Laboratory Animals published by the US National Institutes of Health. The animals were housed on a 12 h light/dark cycle and fed a standard chow diet with water *ad libitum*.

### Treatment with anti-TLR4 antibody

TLR4 inhibition was performed by treating 15 weeks-old SHR and Wistar rats with a daily intra-peritoneal injection of 1 µg of anti-TLR4 antibody (Santa Cruz Biotechnology, USA, sc-13591, rat monoclonal IgG2a, saline-diluted), for 15 days.[12] Control SHR and Wistar rats were treated by the same way with 1 µg of a non-specific IgG antibody (sc-2026 Santa Cruz Biotechnology, IgG2a, saline-diluted) to rule out unspecific effects of TLR4 antibody treatment. In the text the control groups will be identified as IgG or non-specific IgG.

### Arterial blood pressure measurement

Rats were anesthetized with a mixture of ketamine and xylazine (64.9 and 3.2 mg.kg-1, respectively, i.p. injections). The right carotid artery was cannulated with a heparinized polyethylene catheter (PE-50) that was exteriorized in the mid-scapular region. After 24 h, arterial pressure and heart rate were measured in conscious animals by a pressure transducer (model DT-100; Utah Medical Products, USA) and recorded by using an interface and software for computer data acquisition (Quad Bridge Amp/ PowerLab 4/30, ADInstruments, Australia). Heart rate was determined from the interbeat intervals.

### Western Blotting

**Figure 1:**
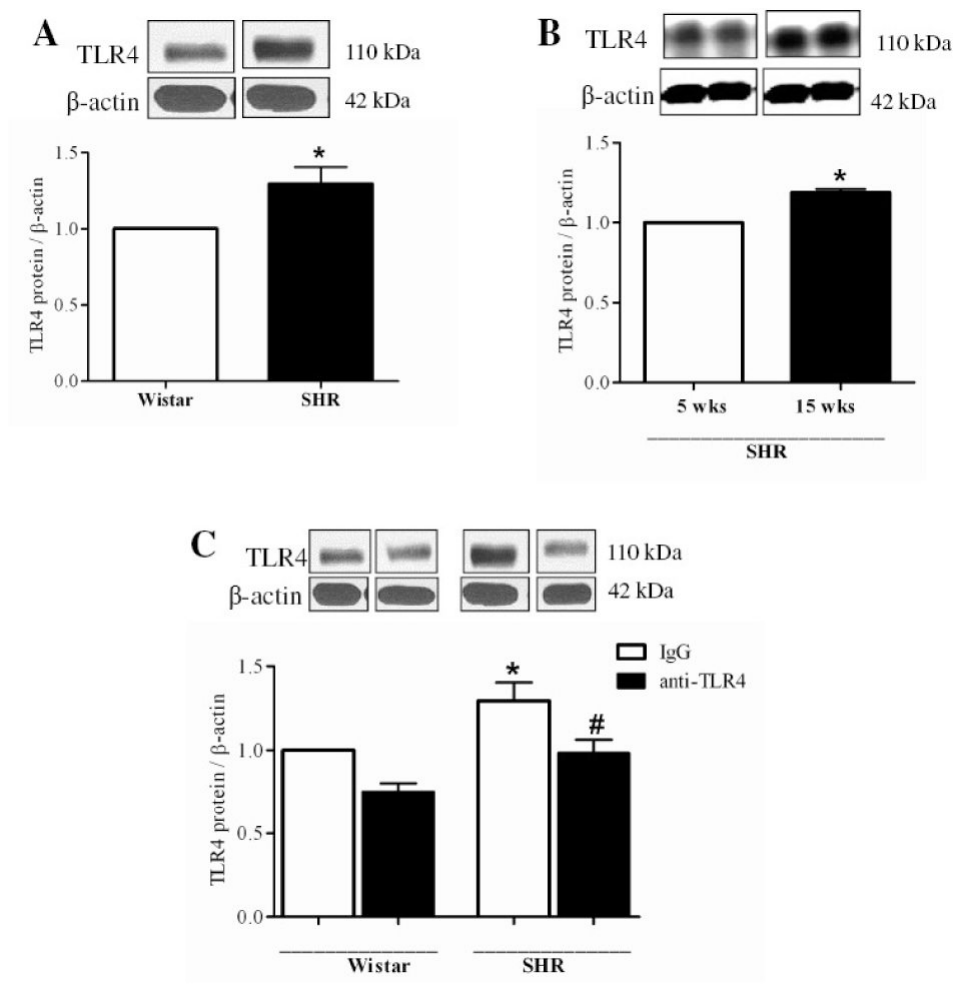
TLR4 protein expression is augmented in mesenteric resistance arteries from 15 weeks-old SHR compared to Wistar with the same age or to SHR 5-weeks-old. Anti-TLR4 treatment decreased TLR4 protein expression in SHR (**A**) TLR4 protein expression was analyzed in mesenteric resistance arteries from 15 week-old Wistar (white bar) and SHR (black bar, and (**B**) in 5 weeks-old (white bar) and 15 weeks-old (black bar) SHR. (**C**) We also evaluated TLR4 protein expression in Wistar and SHR rats treated with IgG or anti-TLR4. On top of A, B and C, representative western blot images of TLR4 and P–actin protein expression. Bar graphs show the relative expression of TLR4 after normalization to β-actin expression. Values are means ± SEM, n= 6. *P < 0.05 compared with Wistar (A), 5 wk-old SHR (B) and Wistar IgG (**C**); # *P* < 0.05 compared with SHR IgG (**C**). Statistical test: Student's t test (A and B) and one-way ANOVA (**C**).

Extracted proteins (50 µg) from small mesenteric arteries were separated by electrophoresis on a 10 % polyacrylamide gel and transferred to a nitrocellulose membrane. Nonspecific binding sites were blocked with 5 % skim milk in Tris-buffered solution (10 mmol/l Tris, 100 mmol/l NaCl, and 0.1% Tween 20) for 1 h at room temperature. Membranes were then incubated with primary antibodies overnight at 4°C. Primary antibodies were: anti-TLR4 (1:250), anti-Cox-1(1:500), anti-Cox-2 (1:500) (all antibodies from Cell Signaling Technology, USA). Membranes were washed with Tris-buffered solution and incubated for 1 hour at room temperature with secondary antibody anti-rabbit (1:1500 dilution). After incubation, membranes were washed with Tris-buffered solution and signals were revealed with chemiluminescence, visualized by autoradiography film, and quantified by densitometry. The same membrane was used to determine β-actin protein expression using a monoclonal antibody against β-actin (1:15000, Sigma-Aldrich, USA), and its content was used to normalize protein expression in each sample. All representative western blot images shown in figures 1 and 6 were obtained from the same membrane.

### Vascular function studies

After euthanasia using isoflurane (via nasal 5% in 100% of oxygen), second-order mesenteric resistance arteries (200-300 µm internal diameter) were removed and cleaned from fat tissue in Krebs solution (in mmol/L: 130 NaCl, 14.9 NaHCO_3_, 4.7 KCl, 1.18 KH_2_PO_4_, 1.17 MgSO_4_·7H_2_O, 1.56 CaCl_2_·2H_2_O, 0.026 EDTA and 5.5 glucose). Arterial segments (2mm in length) were mounted on 40µm wires in a small vessel myograph for isometric tension recording and equilibrated in Krebs solution for about 30 min, gassed with 5% CO_2_ in O_2_ to maintain a pH of 7.4. The relationship between resting wall tension and internal circumference was determined, and the internal circumference, L100, corresponding to a transmural pressure of 100mmHg for a relaxed vessel in situ, was calculated. The vessels were set to the internal circumference L1, given by L1 = 0.9×L100. After stabilization, arterial integrity was assessed by stimulation of vessels, two times with 120mmol/L potassium chloride (KCl). Endothelial integrity was assessed by testing the relaxant effect of acetylcholine (1 µmol/L, Sigma/Aldrich USA) on vessels precontracted with noradrenaline (NA, 3µmol/L, Sigma/Aldrich USA).

Cumulative concentration-response curves to NA (10nmol/L to 100µmol/L, from Sigma/Aldrich USA) were performed in arteries with endothelium. Curves were performed in the presence and absence of either a COX-1 inhibitor (SC-560: 5-(4-chlorophenyl)-1-(4-methoxyphenyl)-3-trifluoromethylpyrazole, 9 nmol/L) or COX-2 inhibitor (NS-398: N-(2-cyclohexyloxy-4- nitrophenyl) methansulphonamide, 10 µmol/L), which were added to the preparation 30 min before starting the concentration-response curves to noradrenaline. Both inhibitors used are from Cayman Chemical, USA.

### Release of Thromboxane B_2_ and 6-keto Prostaglandin F_1α_

Mesenteric arteries were cut into transverse rings 4 mm in length, to measure the release of prostanoids. These were placed for 30 min in siliconized tubes containing 0.5 ml of Krebs solution at 37°C, and stimulated with 100mM of noradrenaline for 15 min. The prostaglandins were measured with a commercially available EIA kit (Cayman Chemical, Ann Arbor, MI). Two-time diluted 50µL samples were used for measurement of thromboxane B_2_ (TXB_2_, stable metabolite of thromboxane A_2_) and 6-keto Prostaglandin F_1α_ (6-keto PGF_1α_, stable metabolite of prostaglandin I_2_). The assays were performed as described in the manufacturer's procedure booklet. The amounts of prostaglandins released are expressed as picograms or nanograms per milligram of wet weight of mesenteric artery.

### Cytokines measure

Serum levels of interleukin-6 (IL-6) and tumor necrosis factor-α (TNF-α) were determined by a quantitative sandwich enzyme immunoassay - commercial ELISA kits (GE Healthcare, USA) in IgG- and anti-TLR4-treated SHR. IL-6 and TNF-α concentrations were expressed as pg/ml.

### Data Analysis

Results are shown as mean ± standard error deviation (SEM) and “n” represents the number of animals used in the experiments. Contractile responses are expressed as the maximum response produced by each agonist concentration. Concentration–response curves were fitted using a nonlinear interactive fitting program (Graph Pad Prism 4.0; GraphPad Software Inc.) and two pharmacological parameters were analyzed: the maximal effect elicited by the agonist (Emax) and the sensitivity to this agonist (-log EC_50_, pD_2_). Statistical analyses of Emax and pD_2_ values were performed using 1-way ANOVA (Post hoc: Tukey) or Student's *t* test, when appropriate. Values of *P*<0.05 were considered statistically significant.

## Results

### TLR4 expression in mesenteric resistance arteries from SHR and Wistar rats

TLR4 protein expression was significantly increased in mesenteric arteries from 15 weeks-old SHR (hypertensive phase) compared to same age Wistar or 5 weeks-old SHR (pre-hypertensive phase) (Figure 1A and 1B, respectively). Treatment of 15 weeks-old SHR and Wistar with anti-TLR4 antibody decreased TLR4 expression in mesenteric resistance arteries from SHR, but not in Wistar rats (Figure 1C). These results demonstrate that TLR4 expression is increased in SHR and that the anti-TLR4 treatment was effective in reducing its expression.

### Effect of anti-TLR4 treatment on hemodynamic parameters and body weight

**Figure 2:**
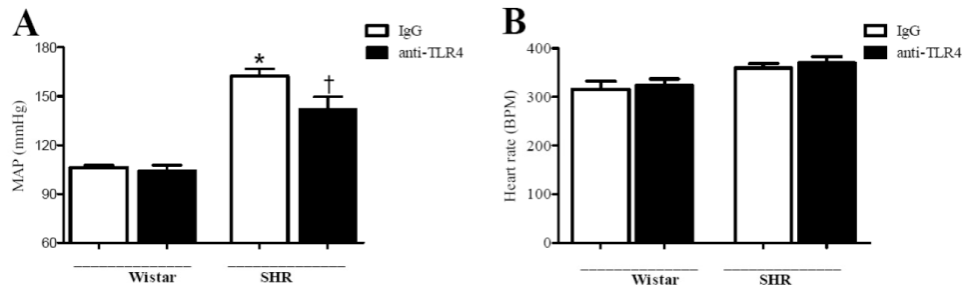
Anti-TLR4 treatment decreased SHR blood pressure (**A**) Mean arterial pressure (MAP, mmHg) and (**B**) heart rate (BPM, beats per minute) were evaluated in Wistar and SHR treated with IgG (white bars) or anti-TLR4 (black bars). Data are expressed as mean ± SEM, n= 6. *P < 0.05 compared with Wistar IgG and #*P* < 0.05 compared with SHR IgG using a one-way ANOVA.

Anti-TLR4 treatment lowered mean arterial pressure in SHR, compared to SHR treated with non-specific IgG (Figure 2A). However, no change in mean arterial pressure was observed in Wistar rats after anti-TLR4 treatment. Heart rate (Figure 2B) was similar between all the groups. Body weight (g) did not change among the groups: Wistar: IgG: 510±15, anti-TLR4: 502±30; SHR: IgG: 334±8, anti-TLR4: 327±13. These results suggest that increased activation of TLR4 contributes to augmented blood pressure observed in SHR.

### Effect of anti-TLR4 treatment on vascular contractility

**Figure 3:**
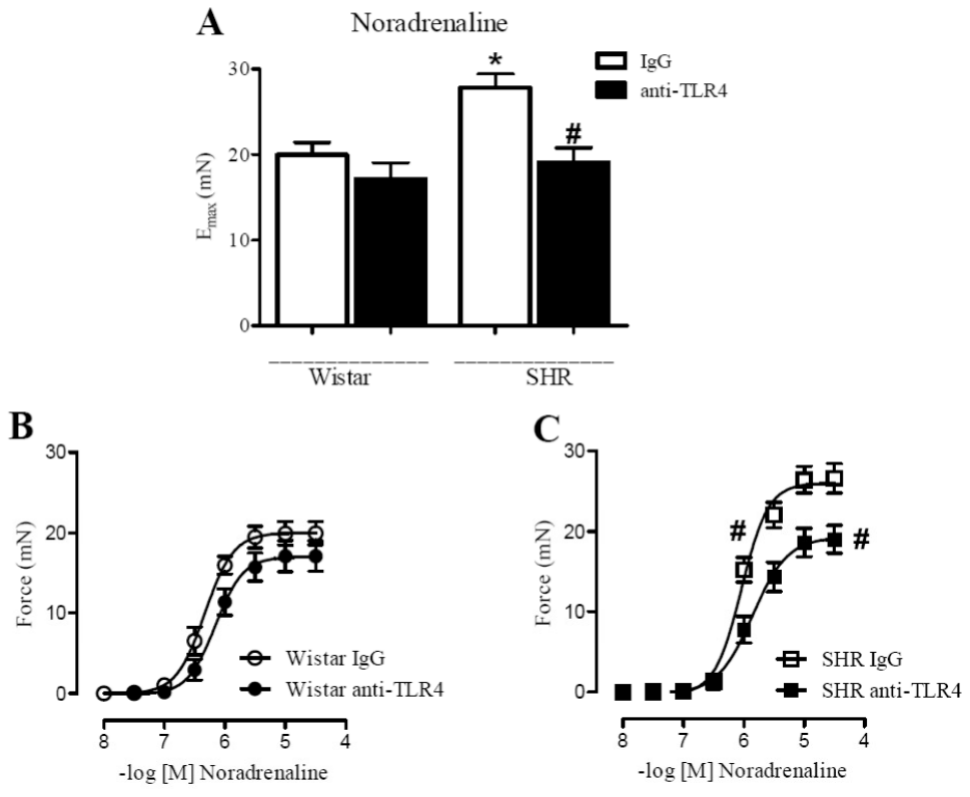
Noradrenaline-induced vasoconstriction in mesenteric resistance arteries from SHR is attenuated by anti-TLR4 treatment Maximal response to (**A**) noradrenaline in mesenteric resistance arteries from Wistar and SHR treated with IgG (white bars) or anti-TLR4 (black bars). Cumulative concentration-response curves to noradrenaline in endothelium-intact mesenteric resistance arteries from (**B**) Wistar treated with IgG (open circle) or anti-TLR4 (closed circle), and (**C**) SHR treated with IgG (open square) or anti-TLR4 (closed square). Each point represents the mean ± SEM of maximal response to each concentration, n = 10-12. *P < 0.05 compared with Wistar IgG and # *P* < 0.05 compared with SHR IgG. Statistical test: one-way ANOVA.

The Emax and pD_2_ to noradrenaline were significantly decreased in endothelium-intact mesenteric resistance arteries from anti-TLR4-treated SHR when compared to those in arteries from unspecific IgG treated SHR (Figure 3A, 3C and Table 1). No differences were observed between arteries from Wistar groups (Figure 3B).

**Figure 4:**
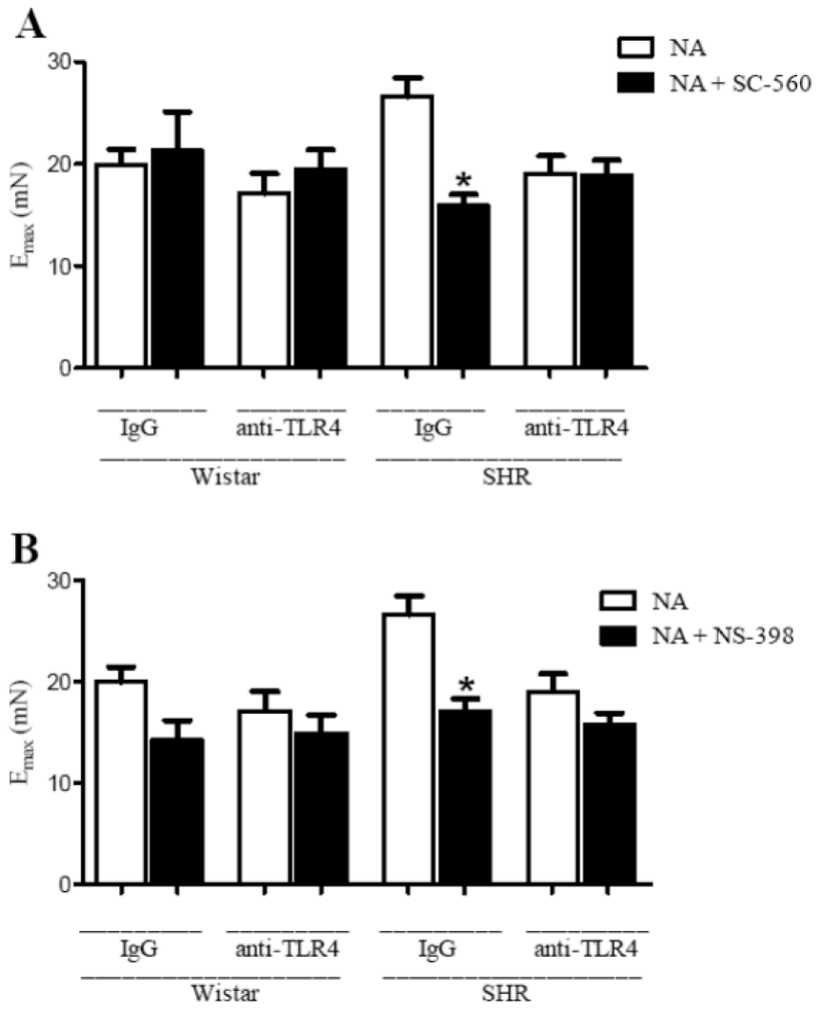
Cox-1 and Cox-2 inhibition decreased the maximal response to noradrenaline only in IgG-treated-SHR mesenteric arteries E_max_ to noradrenaline in the absence (white bars) or presence of (**A**) SC-560 (Cox-1 inhibitor) or (**B**) NS-398 (Cox-2 inhibitor) (black bars) in mesenteric resistance arteries from Wistar and SHR treated with IgG or anti-TLR4. *P< 0.05 vs. SHR IgG NA. Statistical test: one-way ANOVA.

Pre-incubation of mesenteric resistance arteries with either SC-560 (Cox-1 inhibitor) or NS-398 (Cox-2 inhibitor) decreased the Emax and pD_2_ to noradrenaline in arteries from IgG-treated SHR, but not in arteries from anti-TLR4-treated SHR and Wistar groups (Figure 4A and 4B and Table 1).

Together, these data demonstrate that anti-TLR4 treatment decreases the augmented contractile response observed in SHR. In addition, Cox-1 and Cox-2 are signaling pathways that contribute to decreased noradrenaline contraction response in mesenteric resistance arteries from anti-TLR4-treated SHR.

### Effect of anti-TLR4 treatment on Cox expression, pro-inflammatory cytokines and prostanoids release

We evaluated if TLR4 modulates, in SHR, the expression of Cox-1 and Cox-2, as well as the release of the prostanoids TxA_2_ and prostacyclin by mesenteric arteries and the levels of the cytokines IL-6 and TNF-α, in the serum.

**Figure 5:**
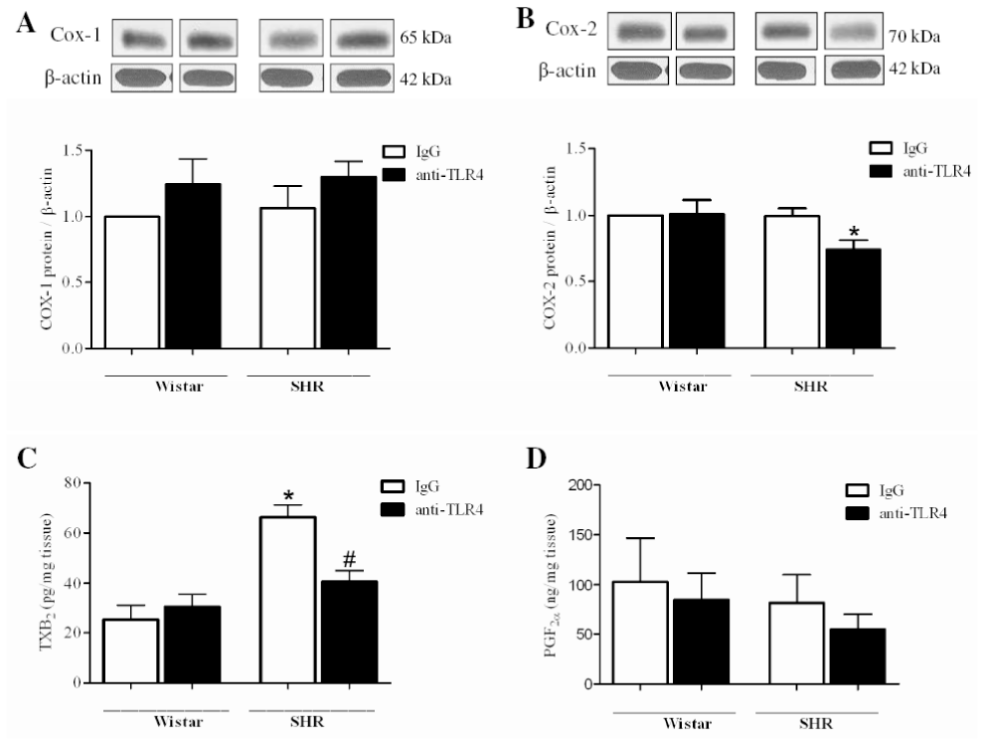
Anti-TLR4 treatment decreases Cox-2 protein expression and TXB_2_ release in mesenteric arteries (**A**) COX-1 and (**B**) COX-2 proteins expression in mesenteric resistance arteries from IgG-(white bars) or anti-TLR4-treated (black bars) Wistar and SHR rats. On top, representative western blot images of Cox-1 (A) and Cox-2 (B) protein expression. Bar graphs show the relative expression of Cox-1 and Cox-2 after normalization to β-actin expression. Release of (**C**) thromboxane B_2_ and (**D**) 6-keto-prostaglandin F_1α_ by mesenteric arteries stimulated with noradrenaline 100 μM. Each bar represents the mean ± SEM, n = 5-6. *P< 0.05. Statistical test: one-way ANOVA.

Cox-1 protein expression in small mesenteric arteries did not change after anti-TLR4 treatment when compared to IgG groups (Figure 5A). However, Cox-2 protein expression was decreased in mesenteric arteries from SHR treated with anti-TLR4 compared to IgG-treated SHR (Figure 5B).

The TxB_2_ released by segments of mesenteric arteries stimulated with noradrenaline (100mM) is increased in SHR IgG when compared with all other groups, and the anti-TLR4 treatment decreased the production of this vasoconstrictor prostanoids in SHR (Figure 5C). No difference was found in 6-keto PGF_1α_ secretion between the groups (Figure 5D).

**Figure 6:**
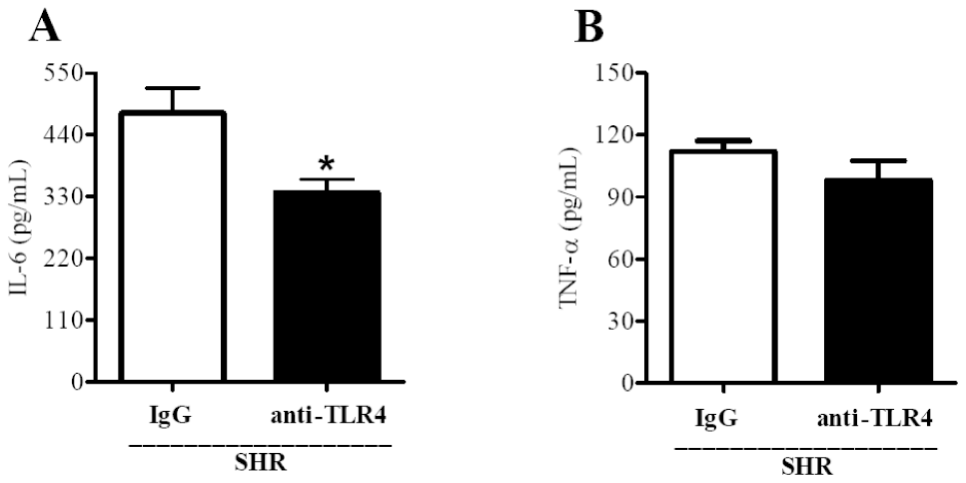
Anti-TLR4 treatment decreases IL-6 secretion in SHR Serum levels of (**A**) IL-6 and (**B**) TNF-α in IgG- (white bars) and anti-TLR4-treated SHR (black bars). Values are means ± SEM, n = 9. *P < 0.05 vs. IgG-treated SHR. Statistical test: Student`s *t* test

Serum levels of IL-6 and TNF-α were measured in IgG-treated and anti-TLR4-treated SHR. IL-6, but not TNF-α level was decreased after treatment with anti-TLR4 in SHR (Figure 6A and 6B).

These data demonstrate that TLR4 inhibition attenuated the activation of inflammatory markers, as demonstrated by decreased Cox-2 expression, lower IL-6 serum levels and thromboxane release in mesenteric arteries from SHR.

## Discussion

The major findings of this study are that the treatment of adult SHR with anti-TLR4 antibody reduces mean arterial pressure as well as decreases contractile responses in mesenteric resistance arteries when compared to non-specific IgG-treated-SHR.

Essential hypertension is characterized by increased peripheral vascular resistance, which is mainly determined by small resistance arteries and arterioles. At the functional level, both an increase in the contractile responses or a decrease in relaxant responses in resistance arteries might increase peripheral resistance. Inflammation of the vascular wall is associated to dysfunction of blood vessels.[13] In view of the decreased blood pressure observed after anti-TLR4 treatment in SHR, we investigated whether this treatment could improve contractile responses in resistance arteries from SHR. Our results demonstrate that mesenteric resistance arteries from anti-TLR4-treated SHR showed lower sensitivity to noradrenaline compared with IgG-treated SHR. However, no changes were found in Wistar groups. These findings suggest that the innate immune response, represented by TLR4, plays a role in the vascular dysfunction associated with hypertension.[13]

Activation of TLR4 results in increase of its own expression.[14] Therefore the reduction of TLR expression is a possible mechanism by which TLR activation can be controlled.[15-16] Our results demonstrated that anti-TLR4 treatment decreased TLR4 protein expression, which is up-regulated in adult SHR compared to young SHR and compared to Wistar rats. The regulation of TLR4 expression was showed in a model of myocardial ischemia/reperfusion, where TLR4 is up-regulated. In those rats, treatment with valsartan, an angiotensin receptor blocker with anti-inflammatory properties, decreased the expression and activation of TLR4.[17]

Anti-TLR4 antibody treatment has been tested in some inflammatory conditions, such as sepsis and chronic bowel disease, with promising results, showing a decrease in the inflammatory process.[18-19] Hypertension is considered as a chronic inflammatory disease, where elevated pro-inflammatory cytokines and Cox-derived prostanoids, mainly from the Cox-2 isoform, are observed.[20] We hypothesized that the reduction in TLR4 effects, by using neutralizing antibody *in vivo*, would ameliorate hypertensive low-grade inflammation. Here we observed that treatment with anti-TLR4 antibody decreased the expression of TLR4, COX-2 and IL-6 serum levels, markers of inflammation. Therefore, our results suggest that TLR4 is associated with low-grade inflammation in hypertension.

The major downstream molecular mechanism involved in TLR4 activation is a MyD88-dependent pathway that involves IL-1R-associated kinases (IRAK), TNF receptor-associated factor-6 (TRAF-6), and mitogen-activated protein kinases (MAPK) and that culminates in the activation of NFκB.[2] In turn, NFκB mediates the transcription of Cox-2 and pro-inflammatory cytokine genes. Kuper et al demonstrated that LPS mediates enhanced Cox-2 expression in renal medullary colletion duct cells by TLR4-mediated activation of the NFkB signaling pathway.[21] In hypertension, as mentioned before, TLR4 expression is increased in the heart of SHR compared to Wistar-Kyoto rats.[6] Conversely, studies such as the one by Li et al showed that NFkB is activated in the heart, kidney and aorta in SHR compared with Wistar-Kyoto rats.[22]

Many studies have shown that TLR4 is clearly responsible for inflammation induced by endogenous ligands, such as C-reactive protein [23] and heat shock proteins (HSP60 and HSP70) in smooth muscle cells and many other cell types.[24-25] In hypertension, these molecules are increased [26-27] and could act as long-term TLR4 activators, resulting in augmented expression of several pro-inflammatory cytokines in vascular smooth muscle.[28] Pro-inflammatory cytokines regulate the expression and function of several proteins, including adhesion molecules, mitogen-activated protein kinases, extracellular matrix components and growth factors, which are important in vessel hypertrophy and vascular dysfunction described in hypertension.[29],[13] Cytokines seem to influence the balance between vasoconstrictor and vasodilator factors, as well as regional differences in the release and responsiveness to these factors, contributing to the increased responsiveness within a specific vascular bed in hypertension.[20]

IL-6 is a pro-inflammatory cytokine that is released from numerous cell types, including endothelial cells [30-31], vascular smooth muscle cells [32], and macrophages [33]. IL-6 stimulates the synthesis of many acute-phase reaction proteins, including C-reactive protein, serum amyloid A, and fibrinogen.[34] IL-6 also promotes vascular smooth muscle cell proliferation, a hallmark of hypertension and atherosclerosis.[35] Some studies have shown a positive association between IL-6 levels and high blood pressure.[36-38] Previous work from Brands et al showed that hypertension caused by high-salt diet and angiontensin II was blunted in mice lacking a functional gene for IL-6.[39] In our study, we observed a reduction in IL-6 secretion in SHR by anti-TLR4 treatment, which was accompanied by a decrease in BP, suggesting that IL-6 is a key cytokine in the development of the pathophysiology of hypertension.

Evidence showed that the vasoconstrictor response to adrenergic agonists is largely mediated by Cox-derived vasoconstrictor prostanoids.[8, 40] Hypertension is thought to modify the role of these products in the vasodilator and vasoconstrictor responses.[8, 41-42] Furthermore, it has been reported that activation of TLR4 by LPS and angiotensin II may induce the release of prostanoids in cultured macrophages and smooth muscle cells.[28, 43] In the present study, we demonstrated that Cox-2 protein expression is decreased in mesenteric resistance arteries from anti-TLR4-treated SHR compared with IgG-treated SHR, and no differences were found in Cox-1 expression among groups. Despite the lack of change in expression of Cox-1, one possibility is that its activity may be decreased after anti-TLR4 therapy. This hypothesis is supported by the fact that inhibition of Cox-1 and Cox-2 reduced the contractile response to noradrenaline only in mesenteric resistance arteries from IgG-treated SHR, while it remained unchanged in resistance arteries from anti-TLR4-treated SHR. In addition, the release of TXA_2_ in mesenteric arteries after noradrenaline stimulation was reduced in anti-TLR4 treated SHR compared with SHR treated with IgG. Taken together, these results suggest that the innate immune response, represented by TLR4 activation, plays a role in the vascular dysfunction associated with hypertension by a Cox-dependent mechanism.[13] Future studies will help clarify the molecular pathways linking these events.

In conclusion, our findings demonstrate that increased TLR4 expression may play an important role in arterial hypertension. Moreover, anti-TLR4 treatment decreased blood pressure, pro-inflammatory mediators and vascular contraction in resistance arteries of SHR.

## Perspectives

Hypertension is an important worldwide public-health challenge because of its high prevalence and concomitant risks of cardiovascular and kidney disease. A variety of pharmacological preparations are available for therapy, however, despite these options, vascular dysfunction persists in many patients, and end-organ injury remains a serious complication. The cardiovascular system is exposed to pathogens and danger signals (endogenous ligands), resulting in activation of pattern-recognition receptors including Toll like receptors. Considering that hypertension is a low-grade inflammatory disease, the innate immune response, represented mainly by TLR, may be investigated as a mechanism that contributes to the development of this condition.

## Figures and Tables

**Table 1:** Maximal response (E_MAX_) and sensitivity (pD_2_) to noradrenaline (NA) with or without inhibitors in mesenteric resistance arteries from Wistar and SHR treated with IgG or anti-TLR4.

	Wistar IgG		Wistar anti-TLR4		SHR IgG		SHR anti-TLR4	

	E_MAX_	pD_2_	E_MAX_	pD_2_	E_MAX_	pD_2_	E_MAX_	pD_2_
NA	19.9±1.4	6.3±0.06	17.1±1.9	6.1±0.08	27.8 ± 1.6†	6.1 ± 0.04	19.0± 1.7*	5.8 ± 0.08*
NA + NS-398	14.2±1.9	6.0±0.07	14.9±1.7	5.9±0.06	17.1 ± 1.3*	5.2 ± 0.05*	15.8 ± 1.1	5.5 ± 0.06
NA + SC-560	21.3±3.7	6.1±0.08	19.5±1.9	6.3±0.07	15.9 ± 1.1*	5.3 ± 0.04*	18.9 ± 1.5	5.5 ± 0.03

Values are means ± SEM. n = 6-12.

* *P* < 0.05 vs. SHR IgG (NA);

† *P* < 0.05 compared with Wistar IgG (NA).

NS-398: Cox-2 inhibitor: SC-560: Cox-1 inhibitor, E_MAX_: maximal response; pD_2_: -log EC50. Statistical test: one-way ANOVA
