# Expression of Concern: Involvement of proteinase activated receptor-2in the vascular response to sphingosine 1-phosphate

**DOI:** 10.1042/CS20130272_EOC

**Published:** 2026-09-10

**Authors:** Fiorentina Roviezzo, Antonella De Angelis, Luana De Gruttola, Antonio Bertolino, Nikol Sullo, Vincenzo Brancaleone, Mariarosaria Bucci, Raffaele De Palma, Konrad Urbanek, Bruno D'Agostino, Angela Ianaro, Raffaella Sorrentino, Giuseppe Cirino

**Affiliations:** 1 Dipartimento di Farmacia, Università di Napoli Federico II, Napoli, Italy; 2 Dipartimento di Medicina Sperimentale, Sezione di Farmacologia L. Donatelli, Seconda Università degli Studi di Napoli, Napoli, Italy; 3 Dipartimento di Chimica, Università degli Studi della Basilicata, Pontenza, Italy; 4 Dipartimento di Internistica Clinica e Sperimentale, Seconda Università degli Studi di Napoli, Napoli, Italy

**Keywords:** aorta, nitric oxide, protease-activated receptor-2 (PAR-2), sphingosine 1-phosphate (S1P), vascular tone

*Clinical Science* has been made aware of potential issues surrounding the scientific validity of this paper and is hence issuing a permanent Expression of Concern to notify readers. A reader has notified us of a concern within the western blots; specifically, the Figure 2C p-Akt and Akt blots (S1P, V and PAR-2AP treatment lanes) appear to be the same as the Supplementary Figure 1 p-Akt and Akt blots (W146, ENMD and V treatment lanes) respectively. The authors were contacted with regard to the concerns but have not responded. As such, the article will remain published but with an associated Expression of Concern.

