# Expression of Concern: Toll-like receptor 4 contributes to blood pressure regulation and vascular contraction in spontaneously hypertensive rats

**DOI:** 10.1042/CS20110523_EOC

**Published:** 2026-09-10

**Authors:** Gisele F. Bomfim, Rosangela A. Dos Santos, Maria Aparecida Oliveira, Fernanda R. Giachini, Eliana H. Akamine, Rita C. Tostes, Zuleica B. Fortes, R. Clinton Webb, Maria Helena C. Carvalho

**Affiliations:** 1 Department of Pharmacology, University of Sao Paulo, Sao Paulo, SP, Brazil; 2 Department of Physiology, Georgia Health Sciences University, Augusta, GA, U.S.A.

**Keywords:** cyclo-oxygenase, hypertension, inflammation, mesenteric resistance artery, Toll-like receptor 4 (TLR4)

*Clinical Science* has been made aware of an instance of high similarity within this article and is hence issuing a permanent Expression of Concern to notify readers. Specifically, it has been noted by a reader that the Figure 1B B-actin bands are identical to each other. During the investigation, the authors explained that the duplication was due to an error during figure assembly.

The reader also highlighted other instances of high similarity; specifically, between:

- The Figure 1A TLR4 bands (lanes 1 and 2) and the 1C TLR4 bands (lanes 1 and 3) respectively.
- The Figure 1A B-actin bands (lanes 1 and 2) and the 1C B-actin bands (lanes 1 and 3) respectively.
- The Figure 1A B-actin band (lane 2) and the Figure 5B B-actin band (lane 3)
- The Figure 5A B-actin bands (lanes 1-4) and the Figure 5B B-actin bands (lanes 1-4) respectively.

The authors clarified that these blots were run on the same membranes, which were then cut and used for multiple figures. The Editorial Board is satisfied with this explanation. The article will therefore remain published but with an associated Expression of Concern to alert readers to the Figure 1B duplication.

